# Supplementary material for: Cortical beta power reflects decision dynamics and uncovers multiple facets of post-error adaptation
Source: Nat Commun. 2018 Nov 28;9:5038. doi: 10.1038/s41467-018-07456-8 (PMC6261941; doi:10.1038/s41467-018-07456-8)
Supplement: Supplementary file 1 — Supplementary Information [file 41467_2018_7456_MOESM1_ESM.pdf]

Cortical beta power reflects decision dynamics and uncovers multiple facets of post-error adaptation

Fischer et al.

Supplementary Note 1

General behavioural effects. General behavioural effects were in accordance with typical results seen in the flanker task. To ensure this, we used logistic single-trial regression to assess task and cognitive control effects on reaction time (RT) and accuracy (main Fig. 1) according to GLMs 1 and 2. RT was higher on incongruent trials (main effect *Incongruence*, $t_{862} = 74.6$, $p = 0$; two-sided t-test against zero, all p-values are Bonferroni corrected) due to stimulus interference, and when participants had less time between their last response and the next stimulus (main effect of *RSI*, $t_{862} = -71.7$, $p = 0$). Erroneous responses were faster than hits (main effect of *Error*, $t_{862} = -30.5$, $p < 10^{-138}$). Interactions *Error* x *Incongruence*, *Previous Accuracy* x *Incongruence*, and *Error* x *Incongruence* x *Distance* in GLM 1 were all significant (ps < 10^{-53}).
The interaction *Error* x *Incongruence* was caused by an accuracy-dependent inversion of the interference effect (main Fig. 1c) and incongruent errors were faster compared to incongruent correct responses. We furthermore found that interference was modulated by stimulus distance (main Fig. 1f,g): RT costs were larger when flankers and target were in closer visual proximity. This finding is compatible with increased response interference when distractors are closer to the target.
Participants overall responded accurately (mean correct >85%). However, on incongruent trials, participants were significantly less accurate (main effect *Incongruence*, $t_{862} = -123.7$, $p = 0$), as was the case in long RSI trials (main effect *RSI*, $t_{862} = -56.6$, $p = 0$). Stimulus *Distance* also interacted with the *Incongruence* of a given trial (main Fig. 1j, $t_{862} = 43.7$, $p < 10^{-220}$), such that accuracy on incongruent, yet not congruent trials, was significantly lower in the *close* condition. This confirms that flanker interference increases RT and decreases accuracy when the distance between target and flanker stimuli is smaller[39].

Dissociating PES, PIA and PERI from conflict adaptation effects. Similar to increases in accuracy following errors, conflict adaptation effects induced by incongruent stimuli on a given trial in the flanker task lead to increases in RT and decreases in error-rates on consecutive trials. Because most errors are committed on incongruent trials, we next tested whether PES and PIA can be explained by conflict adaptation independent of response accuracy or are seen over and above the effects of congruence repetition, specifically on incongruent trials. Therefore, we included a factor that coded for the previous' trial's incongruence (*Previous Incongruence*) as well as the interaction between current and *Previous Incongruence* into the GLM. We ran this regression onto RT on correct trials and again as a logistic regression on accuracy, in order to dissociate PES and PIA. In the RT-GLM, we found that the effect of PES remained unchanged (main effect *Previous Accuracy*, $t_{862} = 48.2$, $p < 10^{-244}$), confirming that PES can be seen over and above conflict adaptation effects. At the same time, there was a main effect of *Previous Incongruence* ($t_{862} = 48.2$, $p < 10^{-244}$) as well as a significant interaction between previous and (current) *Incongruence* ($t_{862} = -66.7$, $p = 0$). The interaction was caused by a repetition of incongruent trials leading to relative behavioural slowing (*ΔRT* +3 ms), compared to a repetition of congruent trials which led to speeding (*ΔRT* -15 ms). Finally, although errors were committed mostly on incongruent trials, the correlation between *Previous Accuracy* and *Previous Incongruence* was relatively low (mean r = 0.22), such that both could clearly be dissociated.
The accuracy-GLM revealed for PIA that inclusion of previous *Incongruence* abolished the main effect for *Previous Accuracy* ($t_{862} = -1.6$, $p = 1$ corrected), while the interaction *Previous Accuracy x Incongruence* remained significant ($t_{862} = 18.1$, $p < 10^{-60}$). To follow up on this, we confined the analysis to incongruent trials but included *Previous Incongruence* into the regression model. Here, PIA was clearly present (main effect *Previous Accuracy* $t_{862} = 19.8$, $p < 10^{-70}$), indicating that neither PES nor PIA can be explained by conflict adaptation itself. As for *Previous Incongruence*, we confirmed that accuracy following incongruent trials increased specifically on consecutive incongruent trials (*ΔAccuracy* +5.6 %, main effect *Previous Incongruence* $t_{862} = -10.4$, $p < 10^{-22}$,

interaction *Previous Incongruence* x *Incongruence* t_{862} = 35.8, p < 10^{-170}). This was not the case on congruent trials that already had a high accuracy of >95% (*ΔAccuracy* -0.01 %). This result was confirmed when the analysis was confined to incongruent trials (main effect *Previous Incongruence* t_{862} = 34.1, p < 10^{-160}).

Post-error changes in BPL are not the result of overlap with preceding error-related activity. We additionally tested if post-error threshold increases depended on the time between previous erroneous response and onset of the next stimulus. The purpose of this analysis was to test whether these post-error effects were time-dependent in the EEG, given their known time dependence on a behavioural level[1], and to exclude that post-error effects seen in BPL can be reduced to overlapping activation caused by previous error trials. In general, this is unlikely, because we found no differences in the baseline and early stimulus processing periods in BPL when we compared post-error to post-correct trials (main Fig. 6a,e; Supplementary Figure 5). However, if BPL changes could be reduced to overlap, we would expect to find changes on short RSI trials alone, but not on long RSI trials. Finally, a reduction of PERI and effects related to PES is to some degree expected, because both effects decrease over time.

To this end, we firstly introduced the interaction between *Previous Accuracy* x *RSI* into the single-trial regression model (GLM 4). We found a weak, but significant, effect here (t_{856} = 3.9, p = 0.001) indicating that BPL-related response threshold increases following errors were stronger on short RSI trials. However, when we confined the model to long RSI trials alone, *Previous Accuracy* remained significant (t_{860} = -7.3, p < 10^{-12}). Therefore, this data speaks against overlapping error-related effects as the only source of the BPL increase at response level. Rather, this data appears compatible with the notion that PES depends on the time between the error and the consecutive trial. This is further confirmed by an additional behavioural test, in which we compare PES between short and long RSI trials. Indeed, PES is lower on long RSI trials (ΔRT short RSI = 52 ms, long RSI = 20 ms, t_{862} = -19.7, p < 10^{-69}), compatible with lower BPL threshold increases.

Correlation between BPL changes and behavioural adaptations. We also tested whether the degree to which response threshold adaptation was reflected in BPL correlated with PES or PIA. We used the regression weight from the threshold analysis for factor *Previous accuracy* and correlated it across participants with PES and PIA. We found a negative correlation for PES (r = -0.094, p = 0.0054), indicating that larger (more negative) regression weights for BPL threshold changes were seen for larger PES. There was no significant effect for PIA (r = -0.06, p = 0.077).

Supplementary Methods

DDM model stages. We assumed that the decision signal in the flanker task undergoes several stages. The start point of each trial was modelled as a baseline period prior to flanker onset that summed to zero, shifted by a free model variance parameter (*sz*) reflecting start points at each trial. This was mainly done because we wanted to obtain predictions of the time-course of the signal comparable to the recorded EEG data. This period was then followed by a second stage for the duration of the non-decision time of each trial, that no longer was zero mean, but could drift randomly away from the start point. In most implementations of the DDM, non-decision time is simply added to the resulting RT (to reduce computational cost), yet this slight variation does not change model predictions. Consecutive to the non-decision time, we assumed that evidence accumulation was driven by flanker direction for as long as these were displayed, and thereafter by the direction of the target stimulus. Finally, the decision signal was modelled to return to baseline as a half-normal distribution with variance 0.1 and start point 1 that downscaled the drift signal once a decision was made, akin to an Ornstein-Uhlenbeck process. This was done as it is unclear if under speeded conditions a decision process truly terminates when a response occurs or continues to evaluate evidence accumulation for a certain period of time to possibly allow quick adjustments.

However, this has no influence on model predictions regarding RT and accuracy and only determines how the diffusion signal behaves *after* a decision has been made which does not influence the start of the next trial.

Model parameters and description. The speed of evidence accumulation in most DDMs is governed by a mean drift-rate *v* and within-trial variance (noise) *s* which scales all other parameters and here was fixed to 0.1. A decision (response) is triggered when the diffusion reaches a criterion (threshold or boundary, black lines with values $\pm a$ in Supplementary Figure 1b). We used symmetrical boundaries which were defined as left-hand responses when the positive boundary was reached first, and as right-hand responses when the negative boundary was reached first. To make the signal comparable to EEG beta power, we flipped all diffusion epochs to reflect the given response as negative. Therefore, in all plots of the diffusion signal, more negative values reflect evidence accumulation in accordance with the selected response option.

While only flankers were on screen, the model's diffusion was driven by the direction of the flanking arrows (i.e., positive when left, negative when right), scaled with a free parameter *f* representing suppression of irrelevant information (< 1) or possibly their amplification (> 1). Thus, the drift rate during the SOA was determined by:

$$(1)\ drift_{SOA} = v_t \times f_t \times fd_t \,.$$

v_t and f_t were determined according to their associated variances (*sv* and *sf*, respectively) per trial and fd_t reflects the direction (+1 or -1) of the flankers. After target onset, the diffusion was governed by the target direction and the regular drift rate v_t alone, i.e., we assume full suppression of flanker direction information once the target is presented. This is based on the completely unambiguous properties of the stimuli used here. This assumption is a simplification and additional parameters that scale a longer-lasting influence of flankers may provide a more realistic model of the task, yet, this simple model did capture the observed behaviour well (see main Fig. 2). Finally, we assumed that despite disappearance of target and flanker arrows after 33 ms of common presentation, the decision continues to form with a constant speed (v_t). This was based on several studies that indicate that constant drift rates account well even for cases in which visual input is masked after a certain period of time[2]. Therefore, left-pointing congruent flankers (Supplementary Figure 1a,b green hand response and drift line) are associated with a positive drift during the SOA and the following target drift. Vice versa, right pointing incongruent flankers are associated with a negative drift during the SOA, but a positive drift thereafter (Supplementary Figure 1a,b orange hand response and drift line). Note that both periods are shifted in time by the non-decision time modelled as a free parameter (T_{er}). T_{er} here simulates the translation time of stimulus evidence into decision formation, that can be expected to mainly reflect visual processing and response mapping, and varies to a certain degree on every trial, for example due to fluctuations of alertness.

Variance parameters. Across trials, it is reasonable to assume that the speed of evidence accumulation (*v*), non-decision time (T_{er}) and the start point (*z*) of evidence (the baseline) vary to some degree. To account for this, we allowed drift rates to vary according to a zero-mean Gaussian distribution with variance *sv*. Those trials with higher drift rates will reach a decision quicker yet may also be more prone to reach the incorrect boundary on incongruent trials, where the flanking stimuli point away from the correct direction. As the task comprised exactly 50% left and right responses, we fixed the mean start point (*z*) of the diffusion process to 0. All models were thus unbiased with regard to the average starting point across the experiment. However, each individual trial's starting point was allowed to randomly vary according to a uniform distribution with lower and upper limits fit as the free parameter *sz* x 0.5. This variance in start points can be interpreted as the range of bias of a participant towards a left- or right-hand response that varies between trials. Additionally, we assumed that the degree to which distractors influence the diffusion process may vary between trials, reflecting selective attention and attention slips. Therefore, we modelled trial-by-trial flanker suppression effects according to a zero-mean Gaussian distribution with variance *sf*. Errors in the model are caused mainly on incongruent trials by a combination of a randomly chosen high starting point bias and consecutive weak flanker suppression (red response hand and drift line in Supplementary Figure 1a,b). We confirm the association between model parameters and behaviour in a separate regression analysis presented in main Figure 3. Finally, we modelled the

non-decision time (T_{er}) single-trial variance with parameter *st* as a uniform distribution with borders *st* x 0.5. This simply reflects that on some trials, evidence may take longer to be processed, for example via fluctuations in attention.

In sum, the full model (DDM 3) comprised up to 4 free parameters (*v*, *f*, *a*, T_{er}) and their trialwise variance (*sv*, *sf*, *sz*, *st*) which were partly fixed for analyses of less complex models (DDMs 1 and 2) which are thus nested within DDM 3. Specifically, DDM 1 and 2 did not include *sf* which was set to 0 here, and in DDM 1 *f* was fixed to 1 such that flankers could not be suppressed.

Model fitting. We fit all models to each individual participants' RT. Human RT was calculated from the beginning of the flanker onset, that is 83 ms before onset of the target, for consistency with the model in order to avoid possibly negative RT (in most flanker tasks, RT is defined from the target onset, yet, this does not affect the shape of the RT distributions). We excluded all trials with RTs below 110 and higher than 1,100 ms overall, as well as the fastest and slowest 1% of responses per subject and additionally all trials where participants responded more than once (i.e., made correction responses) and all congruent error trials for model fitting, as is common practice when fitting sequential sampling models to RT[3]. This procedure removed 57,860 trials, or 6.6% of the sample, and the remaining 880,647 trials were used for model fitting.

For model fitting in all iterations and parameter recovery, we applied the following hard priors which can be seen as boundary parameters. *v* [0.01 - 8.5 upper and lower boundary of flat prior, *sv* [0 - 1.5], *a* [0.01 - 0.45], *sz* [0.05 - 0.3], *ter* [0.1 - 0.4], *st* [0 - 2], *f* [0.1 - 1.5], *sf* [0 - 1.5]. For *f*, we reduced the prior space to [0.1 - 1.1] for fitting of post-error and post-correct trials as we found that almost no subjects were described by values of *f* > 1.

Because closed form analytic solutions for RT distributions of multi-stage DDMs are not readily available (but see [4,5]) and we found the speed of available options unsatisfactory for fitting of this scale of data and additionally because we were mostly interested in the time course properties of the diffusion process, we fit the models using Monte Carlo simulation of the diffusion process with 5,000 simulated trials per iteration. To obtain behaviourally valid models, we split the overall sample of RTs into 10 equal sized quantiles to estimate quantile maximum likelihood statistics[6] (which is similar to a χ^2 statistic) minimising the negative log-likelihood of the observed participant-data given each set of model parameters. The likelihood of each single observed RT is determined by the model's likelihood of predicting an observation in the corresponding bin separated by correct and erroneous responses. Additionally, we used a mixture model assuming 2% outliers uniformly distributed over the space of RTs in error and correct conditions which has been shown to increase model fits[7]. The mean number of correct trials for fitting was 875 ± 66 (SD, range 615 – 1,014), and the mean number of error trials was 145 ± 45 (range 21 - 341), resulting in 755,125 correct and 125,135 error trials. The resulting likelihoods were then log-transformed and scaled with -1 to allow minimisation of negative log-likelihoods summed across all observations. Because this statistic is not a true likelihood, as it relies on data bins, we call resulting BIC values *approximate BIC* (aBIC; akin to [8]).

To determine best fitting parameter combinations, we used differential evolution[9] which we found to outperform particle swarm (Matlab function *particleswarm*) and iterative calls of simplex algorithms (Matlab function *fminsearch*). For individual fits, we used 40 population members and continued fitting until 120 minutes had passed per participant per model (resulting in approximately 10,000 computation hours). Because there is a small amount of stochasticity in the model even with large trial numbers, the best fitting parameter combinations were then evaluated 20 times for all fits, and the parameter set with the lowest negative log-likelihood was therefrom selected for model comparisons and to simulate the decision process for comparison with neural signals. For predictions of the neural signals, we used the mean of the best fitting parameter combinations for each simulated model (DDM 3 in main Fig. 5, DDMs 3.1 and 3.2 in main Fig. 6) with 5.000 simulated trials.

Analyses of model RT and accuracy. We then simulated RT distributions and accuracy for all models compared to all participants which are plotted binned by quantiles in main Figs. 2, 4 and Supplementary Figure 2. For all models, accuracy is much lower on incongruent trials compared to congruent trials comparable to the results

observed in humans (accuracy incongruent 76%, accuracy congruent 96%). For DDM 3, median RT on correct trials was significantly higher on incongruent compared to congruent trials. As in human data, this reversed on error trials where congruent trials had higher RTs than incongruent ones, consistent with separable mechanisms for both sources of errors. The reason for this effect in the DDM is that errors on incongruent trials are caused by low flanker suppression in combination with start point variance randomly favouring the direction of the flanking errors, driving the diffusion across the boundary and leading to fast errors. Congruent errors, on the other hand, are caused by low (or sometimes even negative) drift rates, such that the noise term has a much larger influence over the diffusion process which does not reach boundaries quickly.

Simulation of decision diffusion signals. From the final model we derived the diffusion signal which we split up according to various criteria, for example into fast and slow responses. Model decision processes were locked to the first presentation of flankers in each trial to study baseline effects and the evolution of the signal from this point on and locked to the response in order to investigate the speed of evidence up-ramping towards the response as well as the decision boundary itself.

para-meter	name	DDM 1		DDM 2		DDM 3		DDM 3.1 post-correct		DDM 3.2 post-error	
v	drift rate	2,76	0,09	4,26	0,21	3,74	0,17	2,49	0,08	2,29	0,07
sv	variance in drift rates	0,21	0,03	1,04	0,05	0,93	0,06	0,93	-	0,93	-
a	boundary	0,28	0,01	0,32	0,01	0,37	0,01	0,19	0,01	0,25	0,01
sz	variance in start points	0,25	0,01	0,33	0,02	0,17	0,01	0,17	-	0,17	-
T_{er}	non-decision time	0,30	0,00	0,30	0,00	0,29	0,00	0,30	0,01	0,29	0,01
st	variance in *Ter*	1,42	0,04	1,16	0,05	1,20	0,07	1,20	-	1,20	-
f	flanker suppression	1	-	0,61	0,01	0,53	0,04	0,47	0,02	0,53	0,03
sf	variance in *f*	0	-	0	-	0,63	0,04	0,63	-	0,63	-
s	(fixed) noise of within-trial diffusion	0.1	-	0.1	-	0.1	-	0.1	-	0.1	-
z	(fixed) average start-point bias	0	-	0	-	0	-	0	-	0	-

Supplementary Table 1 - Maximum likelihood parameters of all DDMs.

The table shows the parameters obtained by fitting the models to the individual subject data. DDM 1 included a fixed value for flanker suppression without variance. DDM 2 had flanker suppression as a free factor, and DDM 3 additionally included flanker variance. For the fit to post-correct and post-error trials, variance parameters were fixed to the mean value across the group derived from DDM 3. We compared parameter differences between DDM 3.1 and 3.2 via multiple regression to account for differences in respective other parameters. This revealed that despite the nominal increased in *f* there is indeed a decrease in flanker processing post-errors when accounting for the reduction in *v* and the increase in *a* (main Fig. 4f). Within-trial variance in diffusion noise (*s*) was fixed to 0.1 in accordance with many other studies employing DDMs (e.g., [8]) for all models. This serves as a scaling factor (the model would predict the same RT data when all parameters including *s* double their value). T_{er} can be translated into ms by multiplication with 1000 and its variance *st* by multiplication with 100. Because the task included exactly 50% left- and right-hand responses, we set the bias parameter for start points (*z*) to zero for all models. Individually fit parameters represent mean, the consecutive column shows 99.9% CI.

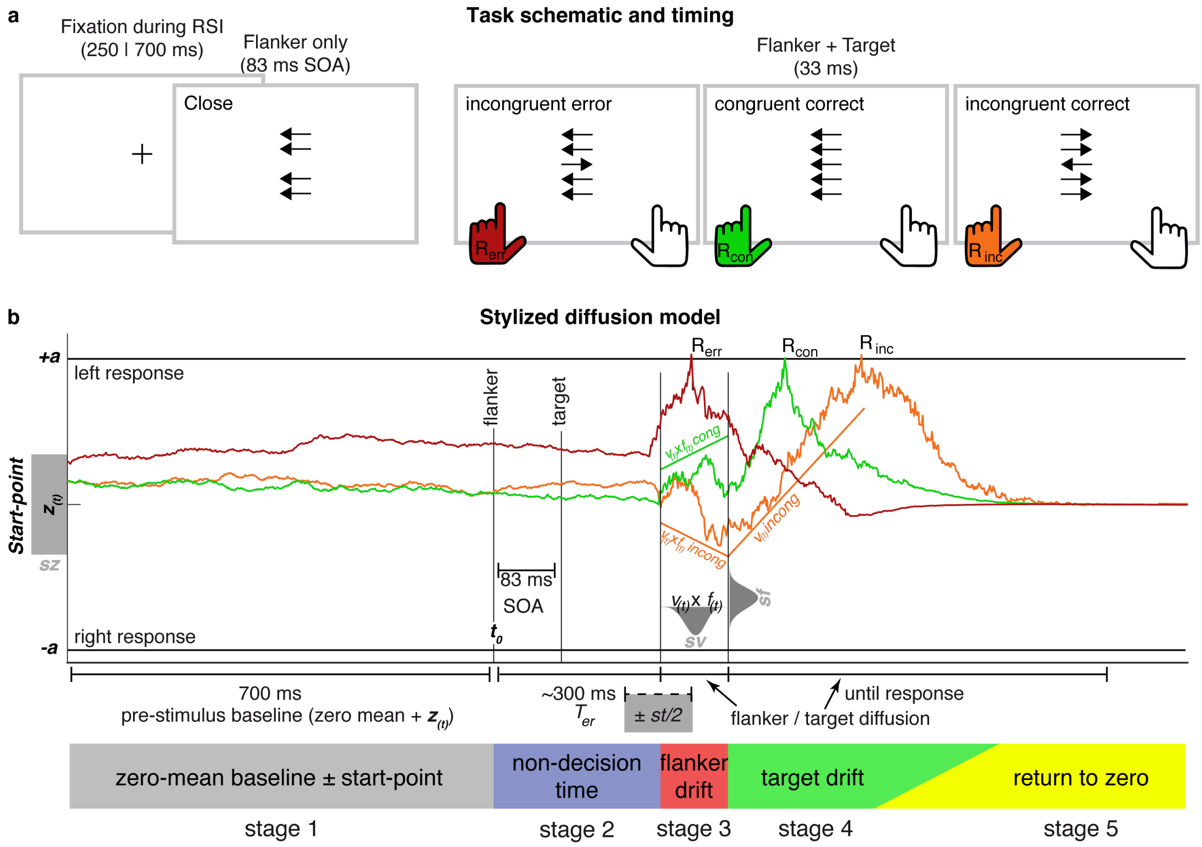

Supplementary Figure 1. Illustration of the multi-stage DDM.

Stimuli in the arrow flanker task (a). At t_0 flankers were presented either closer (second screen) or further away (see main Fig. 1) from the delayed (stimulus-onset-asynchrony (SOA) = 83 ms) target (consecutive screens). The relative direction between flankers and target determines a trial's congruence. Only the target arrow indicates the correct response (green hand = correct congruent, orange = correct incongruent, red = error incongruent). These task phases are explicitly modelled in the DDM (b) with 5 separate stages. This was done as to model the task as closely as possible.

Stage 1 consists of a pre-stimulus baseline for which the value is determined by the start-point variance modelled as a uniform distribution. We did not assume general bias in start points.

Stage 2 models the non-decision time per trial that varies in duration according to the variance parameter *st* again assuming a uniform distribution ($Ter_{(t)} = Ter \pm st \times 0.5$).

Stage 3 represents a noisy diffusion in flanker direction for the duration of the SOA. On incongruent trials (orange), the decision process drifts away from the correct decision with drift rate $v_{(t)} \times f_{(t)}$. The trialwise variance parameters *sv* and *sf* were modelled as Gaussian distributions with mean *v* and *f* respectively. Note that lower values indicate stronger suppression of distractors and $f = 1$ indicates no flanker suppression, whereas values > 1 would indicate stronger flanker compared to target processing.

Stage 4 models the consecutive diffusion back into the correct (left hand) direction with drift rate $v_{(t)}$. Because on incongruent trials the drift in the SOA was opposite to the consecutive drift in stage 4, RTs are longer. Error likelihood increases when the baseline is shifted towards the flanker direction and $f_{(t)}$ is higher on a given trial resulting in an early crossing of decision boundaries (red line). Note that this implies that errors are usually faster on incongruent trials.

Stage 5 models the return to baseline of the decision variable as a half-normal distribution with variance 0.1 and start point 1 that downscaled the drift signal once a decision was made, akin to an Ornstein-Uhlenbeck process. This was done as it is unclear if under speeded conditions a decision process truly terminates when a response occurs or continues to evaluate evidence accumulation for a certain period of time to possibly allow quick adjustments. However, this has no influence on model predictions regarding RT and accuracy and only determines how the diffusion signal behaves *after* a decision has been made which does not influence the start of the next trial.

Grey shades illustrate the distribution of trial-by-trial variance parameters (*sz* = start point variability, *sv* = drift rate variability, *sf* = flanker suppression variability, *st* = non-decision time variability). $\pm a$ = upper / lower decision boundary, $z_{(t)}$ = start-point per trial, $v_{(t)}$ = drift rate per trial, $Ter_{(t)}$ = non-decision time per trial, $f_{(t)}$ = speed of flanker relative to target diffusion per trial. $R_{err,con,inc}$ = time of error, congruent and incongruent response.

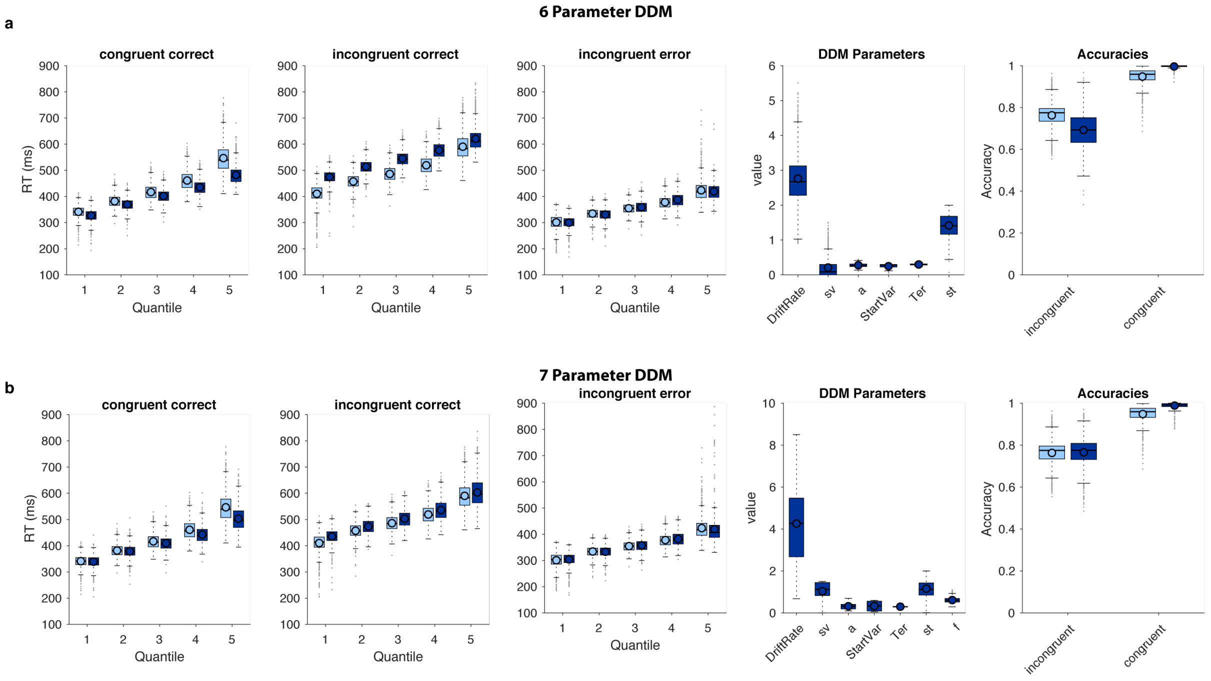

Supplementary Figure 2. Fit for alternative DDMs.

Neither a one-stage DDM with all free variance parameters (a), nor a DDM with an additional flanker suppression parameter that did not vary from trial-to-trial (b) could fit the data as well as DDM 3 that included trialwise variance in flanker suppression. While both models accurately matched error RTs (middle column), they underestimate RT on congruent trials. Especially the model without a flanker parameter (a) severely overestimates RT on incongruent trials. Additionally, this model predicts too many errors overall, yet too few errors in the congruent condition. The reason why the model cannot fit RT and error rates at the same time is that without distractor suppression especially those individual trials are likely to lead to error responses that display high drift rates in the incongruent condition. When incongruent trials with high drift rates cause errors, this leads to selective selection of low-drift rate trials for correct incongruent trials which thus are slower. Thus, adding variance (*sf*) to the flanker suppression parameter (*f*) increased model accuracy (see main Fig. 2b).

Plot conventions as in main Figure 2, all fits were derived from all trials available per subject but excluded congruent errors, as in the fit for the main model.

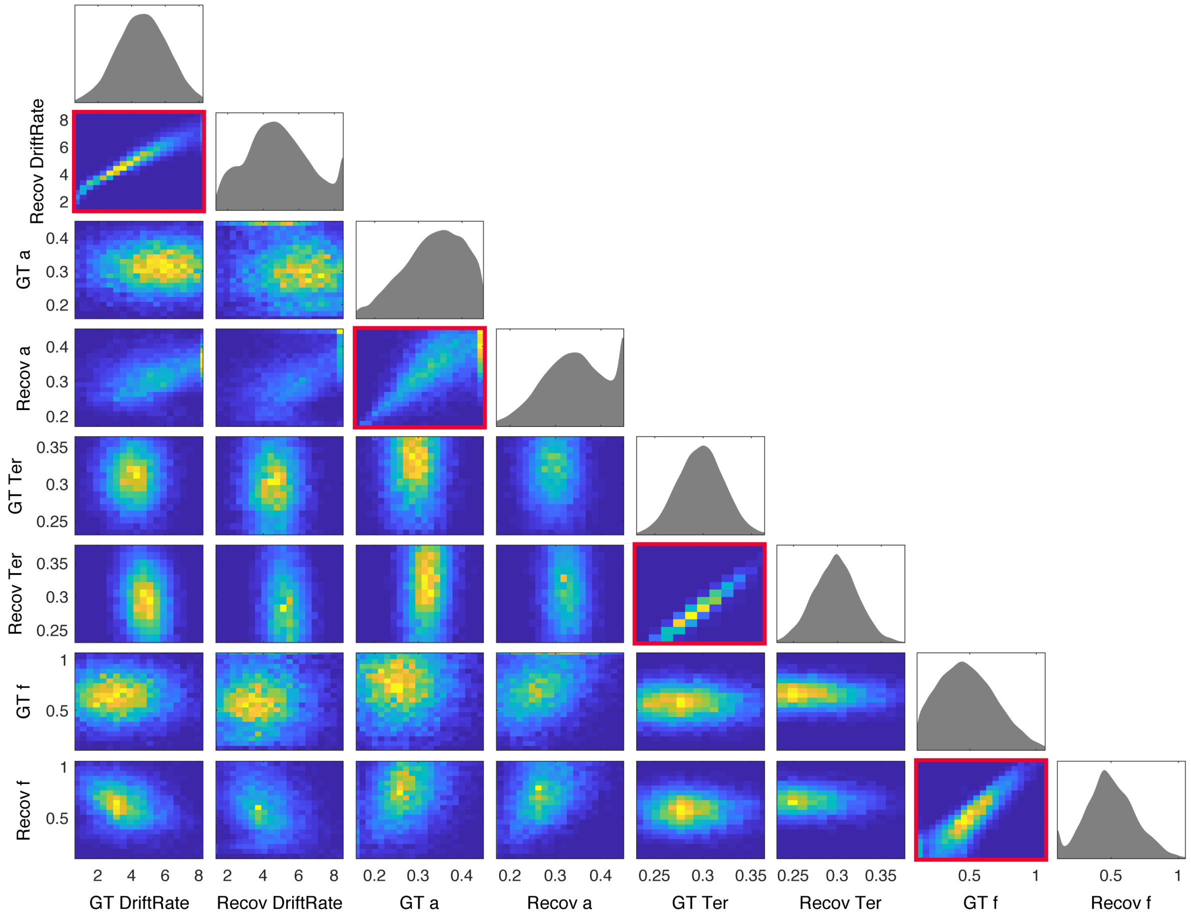

Supplementary Figure 3. Parameter recovery.

Because we compare parameters between two separate DDMs that were fit to post-error and post-correct trials, we also tested if our fit procedure could recover parameters. Therefore, we randomly drew model parameters out of a Gaussian distribution with mean and variance equal to the observed fitted parameters across the whole group to reduce parameter value combinations that were extremely unlikely to occur in human data. We simulated 10.000 parameter combinations and used the same differential evolution algorithm to recover the fitted models. Models that produced no errors at all or for which constraints were not met, were discarded from analysis. As in the human data, we used 5.000 trials per simulation.

Plotted are ground truth (GT) parameters against the recovered parameters (plots marked in red borders). Additionally, we display the whole space of parameter combinations (maps without red borders) and marginal posterior distributions for each of the GT and recovered parameters (k-means smoothed distributions).

Recovered parameters for drift rate (v), non-decision time (T_{er}), and flanker suppression (f) closely resembled the GT parameters over the whole space of possible values. Very low f values could not be recovered, likely because differences in this lower value range did no longer affect accuracy and overall fits were poor for these values. Boundary parameter (a) was well recovered up until the extremer values when recovery sometimes got stuck with the boundary value and high boundaries were less well recovered. Still, there was a clear correlation between GT and recovered parameters. There was some dependency between parameters as well. The only clear association between parameters, which ideally should be completely orthogonal (reflected as round plots), was that when boundary had large values, f could not be recovered. The reason for this is that in such cases the flankers never drive the decision variable across the boundaries and therefore cannot influence RT and error rate significantly.

Overall, this simulation indicates that the model we used to investigate DDM parameter differences in a dual-stage DDM including distractor suppression was capable of detecting true parameter differences well.

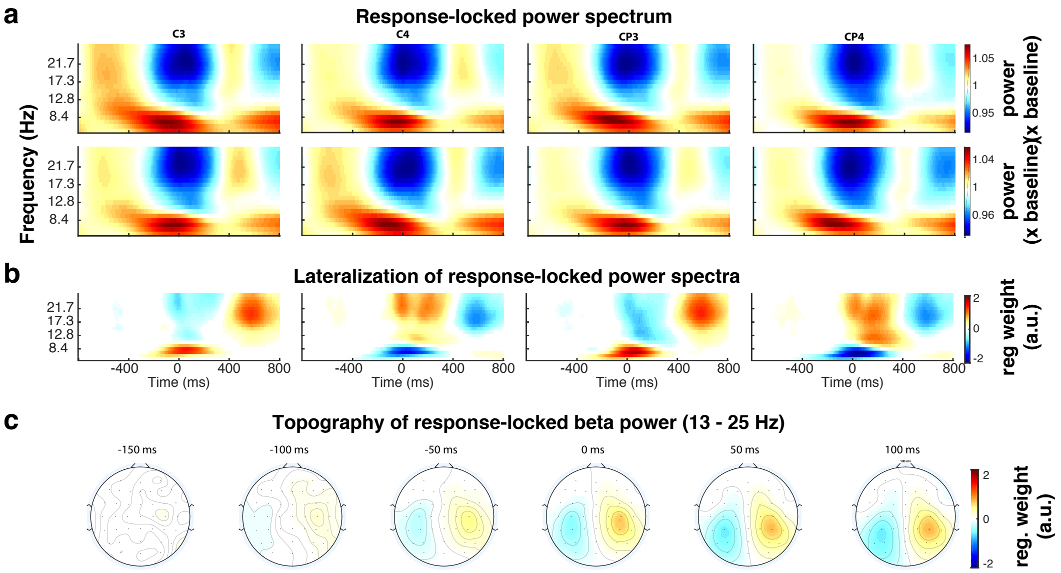

Supplementary Figure 4. Beta power and beta power lateralisation.

a) shows frequency dependent power changes at lateral electrodes around the onset of a response for left (top row) and right (bottom row) responses, confirming that beta power decreases over both hemispheres whenever a response is given. b) shows the result of a single-trial regression analysis comparing responses with the left and right hand, thus reflecting the difference between both responses. Beta power more strongly decreases when a response is given with the contralateral compared to the ipsilateral hand. The topography of this regressor's regression weights (c) confirms maximal effects at electrodes C3/4 and CP3/4. Collapsing over this beta signal and alignment to the effective contralateral site shows the time course of the beta lateralisation signal.

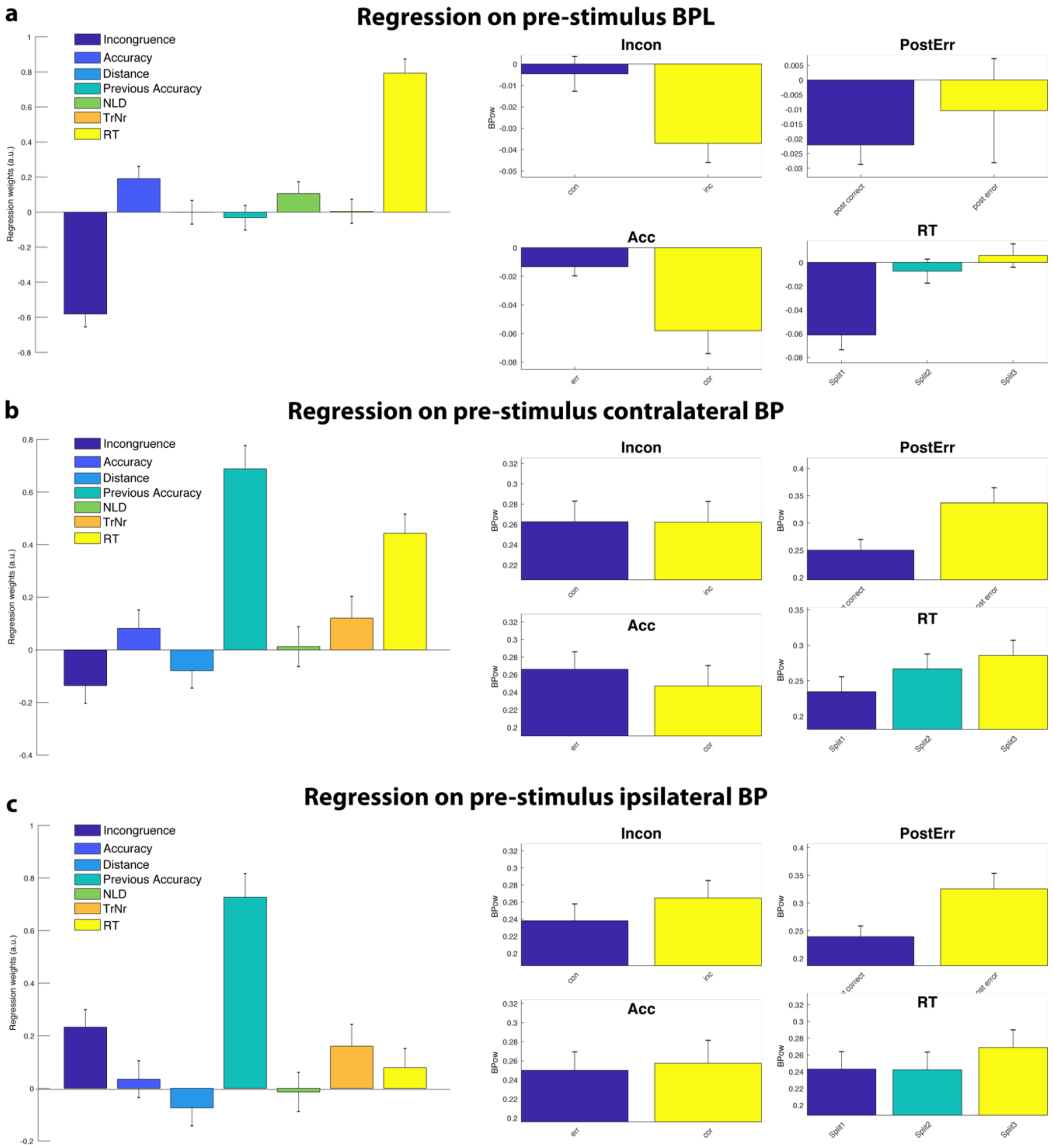

Supplementary Figure 5. Single-trial regression on pre-stimulus beta power.

Because we found that the DDM decision variable predicted plausible influences of the state of the decision in the baseline period and during the random noise that was not driven by any stimulus input, we looked more specifically for ipsilateral, contralateral and BPL associations before stimulus onset (-100 until 0 ms around flanker onset). We used the same regression as in main Figure 6 extended by the current trials (future) accuracy. For post-error effects, we found that BPL was unchanged (p = 1 corrected), whereas beta power over ipsi- (t_{862} = 15.9, p < 10^{-48}) and contralateral hemispheres (t_{862} = 15.1, p < 10^{-44}) was strongly increased following errors which demonstrates that global, yet not BPL, unspecifically increases following errors. Interestingly, while ipsilateral BP (t_{862} = 2.1, p = 0.25) was not significantly related to RT, contralateral BP (t_{862} = 11.9, p < 10^{-28}) showed positive associations with RT (stronger BP was followed by higher RT), BPL was most strongly associated with the RT of the upcoming choice (t_{862} = 19.2, p < 10^{-66}). Moreover, while neither ipsi- (p = 1) nor contralateral (p = .15) BP were predictive of a trials' accuracy, BPL was (t_{862} = -6, p < 10^{-23}). Note that the significant effect of *Incongruence* does not indicate a spurious result but is a result of mapping data to the given response. It indicates that even before stimulus onset the association between the later selected response and BPL is stronger in incongruent compared to congruent trials (t_{862} = -15.6, p < 10^{-47}). The same is seen in the DDM, where start-point variability influences decisions more strongly when the trial is incongruent as the decision boundary is reached early during the time of flanker processing. These results demonstrate that BPL is a measure that cannot be reduced to the simple analysis of both hemispheres alone because neither was associated with upcoming accuracy.

All follow-up plots show raw BP / BPL data (insets on the right), error-bars reflect 99% CI for regression weights, and 99.99% CI for raw data. Results are adjusted for multiple comparisons via Bonferroni correction. NLD reflects the log-distance from the last break in the task, and trial number the general time on task.

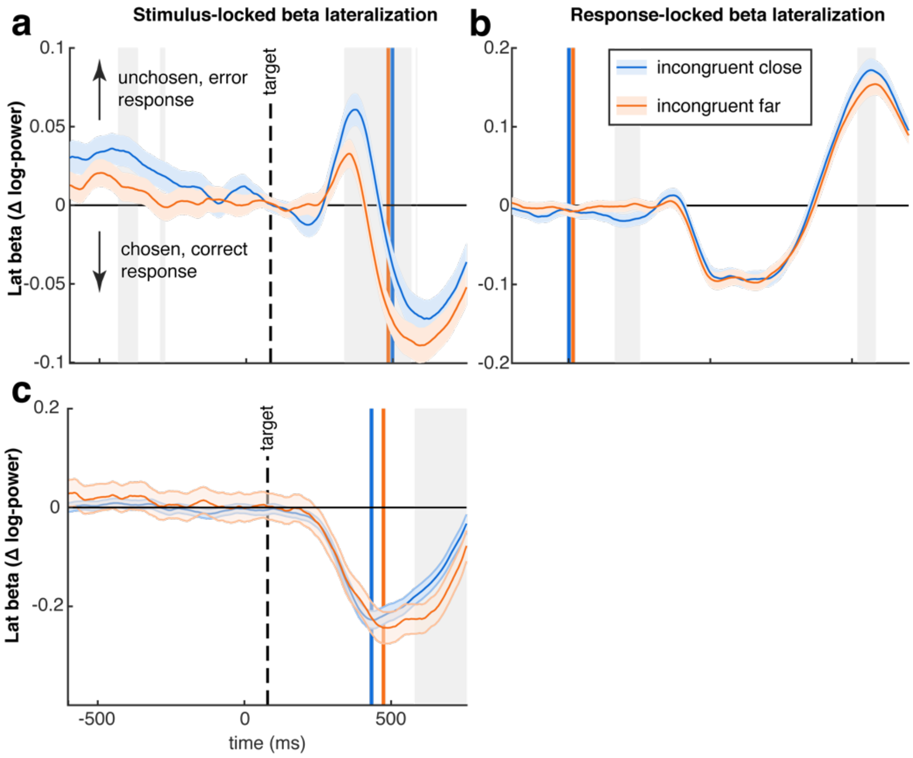

Supplementary Figure 6. BPL reflects the degree of trial-wise conflict.

We additionally separated all incongruent trials by whether the distractors were presented in close visual proximity (blue) or further away from the target stimulus that had to be attended (orange). Plots display BPL time courses locked to stimulus onset (a) and response execution (b) We found that when distractors were closer to the target stimulus, BPL was more driven in the direction of the distractor. This confirms that BPL is sensitive to conflict induced on a trial-by-trial basis.

(c) Shows the stimulus locked processing of congruent trials following correct and error responses, for comparison with incongruent trials presented in main Figure 7e. Additionally, like in the slope analysis, there is no significant difference in processing congruent information depending on the accuracy of the previous trial apparent.

Note that we aligned both conditions from 0 to 200 ms following stimulus onset to account for the lateralisation induced by selecting only correct trials (main Fig. 5f).

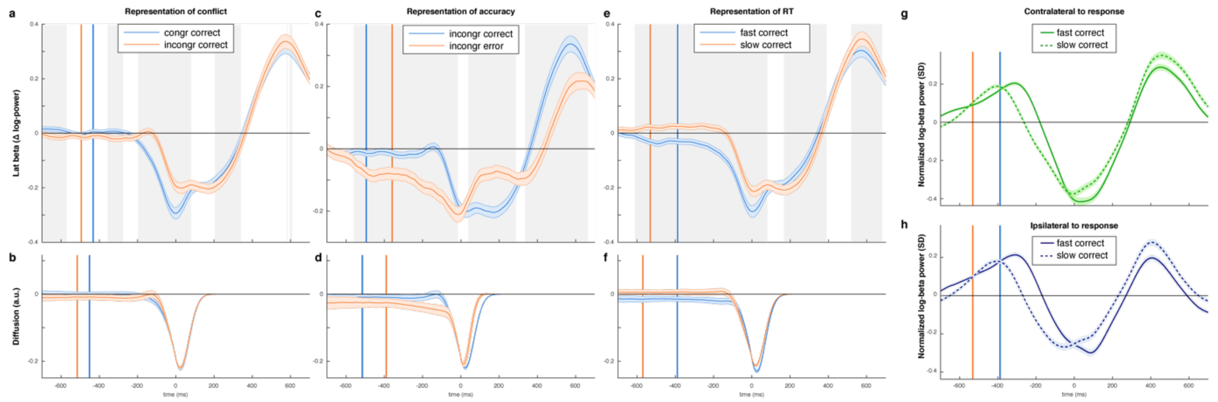

Supplementary Figure 7. Response-locked BPL and DDM decision variable.

Akin to main Figure 5, that displays stimulus processing for trials separated by stimulus congruence, accuracy and response speed, we display the same trials here when the analysis is locked to the response. In the BPL signal when comparing correct congruent and incongruent trials (a), we found a significant difference at response execution. Our follow-up analysis of the lateralised signal showed that this effect is due to reduced activation (increased beta power) of the ipsilateral hemisphere on congruent trials compared to incongruent ones. This effect is not modelled in the DDM (b) because it does not include threshold differences between trials.

When comparing correct and error responses, we note that both are associated with similar thresholds in BPL (c) which is comparable to the modelled DDM signal (d). At virtually all other times in the trial, BPL differs between correct and error responses.

As in the stimulus-locked analyses, we find that in BPL (e) as well as modelled signal (f) a lateralisation towards the given response is pronounced for fast responses. Notably, BPL at response execution is reduced for later responses. If BPL is seen as a reflection of response thresholds, this observation would be compatible with dynamically changing decision bounds within a trial [10].

This effect is partly explained by a reduction of beta power over the contralateral hemisphere (g), while on average beta power over the ipsilateral hemisphere is barely changed when responses are given later in a trial (h). Yet, BPL showed a stronger influence of response time than either of the hemispheres alone suggesting that it may be an especially valid marker of response threshold modulations (contrast contralateral against BPL effect $t_{862} = 5.3$, $p < 10^{-6}$, main effects contralateral $t_{862} = 5.1$, $p < 10^{-5}$ and ipsilateral $t_{862} = -3.6$, $p < 10^{-3}$).

Note that our diffusion model did not simulate beta rebound (the lateralisation of beta power following response execution). Thus, the model immediately returns to baseline after a decision is made. Plot conventions as in main Figure 5.

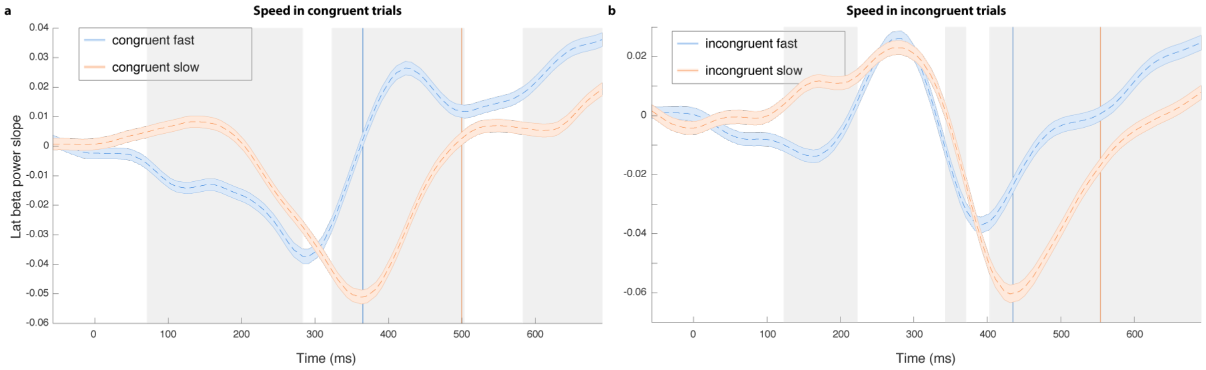

Supplementary Figure 8. BPL slope for fast and slow trials.

Akin to main Figure 8, that displays stimulus processing for trials separated by conflict, degree of interference and post-error effects, we display here how response speed is reflected in the BPL slope signal. On congruent (a) as well as incongruent (b) trials, slower responses are preceded by very early slopes hinting in the direction of the response that will not be selected later in the trial (vertical lines represent mean RT per condition). Slope changes induced by incongruent trials (b) are comparable during the time distractors are processed, but more quickly gain in steepness on fast trials (effect 340 to 380 ms). Interestingly, on congruent trials BPL slopes have returned to baseline when responses are made, whereas they continue to unfold in the incongruent condition.

Grey areas reflect significant time-points of paired *t*-test between conditions following Bonferroni correction, shades reflect SE.

Supplementary References.

1. Danielmeier, C. & Ullsperger, M. Post-error adjustments. *Front. Psychol.* **2,** 233 (2011).
2. Ratcliff, R. & Rouder, J. N. A diffusion model account of masking in two-choice letter identification. *Journal of Experimental Psychology: Human Perception and Performance* **26,** 127–140 (2000).
3. Vandekerckhove, J. & Tuerlinckx, F. Fitting the ratcliff diffusion model to experimental data. *Psychon Bull Rev* **14,** 1011–1026 (2007).
4. Diederich, A. & Oswald, P. Multi-stage sequential sampling models with finite or infinite time horizon and variable boundaries. *Journal of Mathematical Psychology* **74,** 128–145 (2016).
5. Srivastava, V., Feng, S. F., Cohen, J. D. & Leonard, N. E. A martingale analysis of first passage times of time-dependent Wiener diffusion models. *Journal of Mathematical Psychology* (2016). doi:10.1016/j.jmp.2016.10.001
6. Heathcote, A., Brown, S. & Cousineau, D. QMPE: estimating Lognormal, Wald, and Weibull RT distributions with a parameter-dependent lower bound. *Behav Res Methods Instrum Comput* **36,** 277–290 (2004).
7. Wiecki, T. V., Sofer, I. & Frank, M. J. HDDM: Hierarchical Bayesian estimation of the Drift-Diffusion Model in Python. *Front Neuroinform* **7,** 14 (2013).
8. White, C. N., Ratcliff, R. & Starns, J. J. Diffusion models of the flanker task: Discrete versus gradual attentional selection. *Cognitive Psychology* **63,** 210–238 (2011).
9. Chakraborty, U. K. *Advances in Differential Evolution*. **143,** (Springer Science & Business Media, 2008).
10. Hawkins, G. E., Forstmann, B. U., Wagenmakers, E. J., Ratcliff, R. & Brown, S. D. Revisiting the Evidence for Collapsing Boundaries and Urgency Signals in Perceptual Decision-Making. *Journal of Neuroscience* **35,** 2476–2484 (2015).
